# HAT-field: a cheap, robust and quantitative Point-of-care serological test for Covid-19

**DOI:** 10.1093/biomethods/bpac026

**Published:** 2022-11-28

**Authors:** Etienne Joly, Agnès Maurel Ribes

**Affiliations:** CNRS, Institute of Pharmacology and Structural Biology (IPBS), University of Toulouse, Toulouse 31000, France; Laboratoire d’Hématologie, Centre Hospitalier Universitaire de Toulouse, 31000 Toulouse, France

**Keywords:** quantitative, IH4-RBD, haemagglutination, Covid-19, SARS-Cov-2

## Abstract

The haemagglutination test (HAT)-field protocol described here is an optimization of the recently published HAT, for the detection of antibodies directed against the receptor binding domain (RBD) of the SARS-Cov-2 virus. HAT and HAT-field are both based on haemagglutination triggered by a single reagent, the IH4-RBD recombinant protein. A sample of IH4-RBD sufficient for several thousand tests or a plasmid encoding IH4-RBD can be obtained from the authors of our first paper. Using titration of IH4-RBD, HAT-field now allows a quantitative assessment of antibody levels in a single step, using a few microliters of whole blood, such as can be obtained by finger prick, and requires only very simple disposable equipment. Because it is based on a single soluble reagent, the test can be adapted very simply and rapidly to detect antibodies against variants of the SARS-CoV-2, or conceivably against different pathogens. HAT-field appears well suited to provide quantitative assessments of the serological protection of populations as well as individuals, and given its very low cost, the stability of the IH4-RBD reagent in the adapted buffer and the simplicity of the procedure, could be deployed pretty much anywhere, including in the poorest countries and the most remote corners of the globe.

## Introduction

For the past 2 years, the Covid-19 pandemic has preoccupied the whole world, and it remains a major concern for all nations, albeit with different perspectives depending on their wealth. In affluent nations, most people have now been vaccinated and the main issues now are when to start offering booster vaccinations and to whom. Poorer countries, by contrast, have had limited access to vaccines or even to diagnostic tests simply to follow the progress of the pandemic within their populations. To date, most tests available to monitor immune responses against SARS-CoV-2 either require elaborate laboratory procedures and equipment, or are not sufficiently sensitive or quantitative to be of real value [1–3]. For both affluent and less affluent countries, access to a robust and reliably quantitative point-of-care (PoC) serological test would be a great asset to tackle these problems. Such a test would allow health professionals, and health authorities, to distinguish people with either no or waning levels of antibodies, who should have priority for vaccination or re-vaccination, from those with high levels of antibodies against the SARS-CoV-2 virus, who may not need to be vaccinated or revaccinated immediately, and may actually be the ones most likely to suffer undesirable effects from vaccine injections.

Last year, we described a very simple, inexpensive serological test for Covid-19 called the haemagglutination test (HAT) [4]. HAT uses a recombinant protein (IH4-RBD) comprised of a nanobody, IH4, which binds to human glycophorin at the surface of red blood cells [5], fused to the receptor binding domain (RBD) of the SARS-CoV-2 virus. When mixed with diluted human blood, this reagent coats the RBCs and, if antibodies to the viral RBD domain are present in the blood sample, they will cause haemagglutination. This test thus detects specifically antibodies against the RBD, which means that it can be used as a surrogate sero-neutralization test since those antibodies are the main ones endowed with sero-neutralizing activity against the virus [6–8]. Another important feature of HAT is that, because it is based on a soluble reagent, it can be adapted very easily and rapidly to detect antibodies against different variant forms of the virus [6, 7] or presumably to other pathogens if needed be, for example, in the context of a newly arising pathogen.

In the format initially described for HAT, quantitative evaluation of the levels of antibodies was possible via serial dilutions of serum or plasma before mixing with washed autologous RBCs, or obtained from O-donors [4]. In its simple single-point format, HAT was recently used to measure seropositivity rates in Sri Lanka and compared well with a sensitive ELISA [9]. Here, we describe an adapted protocol, called HAT-field, which is quantitative through titration of the IH4-RBD reagent and can be performed in a single simple step with no specialized equipment. The observation that the performances of the assay are minimally affected by temperatures and that, in the optimized HAT-field buffer, which contains BSA and azide, the reagent is stable for weeks with no refrigeration required could also greatly facilitate the use of HAT-field in remote locations.

## Results

### BSA prevents adsorption of IH4-RBD to the reaction wells

Following the method originally described by Wegmann and Smithies [10], the original HAT protocol uses 96 conical-well plates [4]. When appropriately diluted blood is mixed with the IH4-RBD reagent in these conical wells, the RBCs sediment during the incubation of 60 min; haemagglutination due to specific antibodies against RBD in the blood is observed by the formation of persistent ‘buttons’ of RBCs in the bottom of the well when the plate is tilted, whereas in the absence of haemagglutination a ‘teardrop’ shape forms [4].

**Figure 1:**
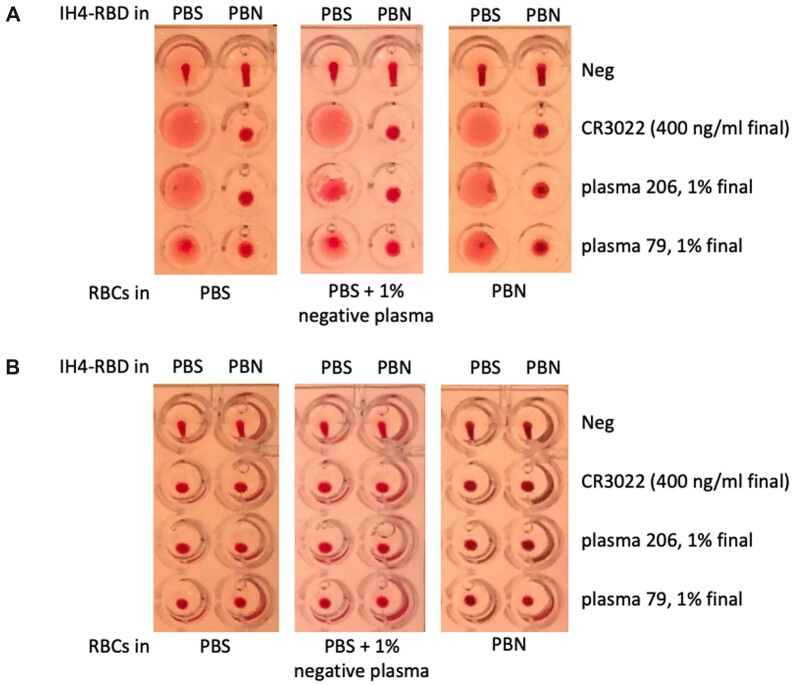
BSA prevents adsorption of IH4-RBD to the reaction wells. (**A**) HAT was performed in uncoated wells prefilled with IH4-RBD reagent diluted in either PBS (left columns) or PBS + 1% BSA + 3mM sodium azide (PBN, right columns). RBCs, resuspended in either PBS (left panels), in PBS supplemented with 1% seronegative autologous plasma (middle panels) or PBN (right panels) and various antibodies against SARS-CoV-2 were added to each well to test for haemagglutination. In the absence of antibody (Neg), the typical teardrop structure can be seen in each well. In the presence of a monoclonal antibody against RBD (CR3022), or plasma from convalescent Covid-19 patients, a veil structure forms in the absence of BSA, whereas a button forms when BSA is present (see the ‘Methods’ section for details). B, HAT as in (A) but performed in wells precoated with BSA. In the presence of the monoclonal antibody or convalescent patient plasma, haemagglutination is observed as a button rather than the veils seen in (A). Similar data were obtained from three experiments.

To perform HAT in field conditions and/or on large numbers of samples, it would be much simpler for the users to be provided with the IH4-RBD reagent already distributed in the V-well plates used to perform the HAT tests. The plates, however, are made of polystyrene, to which many proteins tend to adsorb. (This non-specific adsorption is the basis for many ELISA tests [11].) Indeed, when the IH4-RBD reagent was diluted in PBS and placed in the wells, some of it was readily adsorbing to the plastic of the wells’ slopes, and causing the formation of diffuse veils in haemagglutinated wells, which not only raised concerns about losing some of the active reagent by its immobilization on the plastic, but could make the reading of the results of the HAT tests less clear than the formation of bright red buttons (Fig. 1A). Furthermore, in preliminary experiments involving serial dilutions of the IH4-RBD reagent, we found that the diluted IH4-RBD reagent tended to be lost rapidly through this phenomenon of adsorption.

We solved this problem of veil formation by diluting the IH4-RBD reagent in PBN (Fig. 1A, right columns of each panel), which is Phosphate Buffer Saline (PBS) containing 1% Bovine Serum Albumin (BSA) and 3 mM sodium azide (NaN3), to prevent contamination by micro-organisms. In PBN, rather than veils, buttons of haemagglutination were observed and could be distinguished easily from the teardrops in the negative controls. These buttons formed whether the RBCs, which were added after the IH4-RBD reagent, were resuspended in PBS, in PBS supplemented with 1% plasma from the same seronegative donor as the RBCs, or in PBN. We conclude from this experiment that veil formation is due to adsorption of the IH4-RBD reagent to the polystyrene walls of the wells. This interpretation is supported by our finding that no veils formed when the wells were precoated with BSA and rinsed with PBS before performing the assay (Fig. 1B).

### Azide and BSA increase the sensitivity of HAT

**Figure 2:**
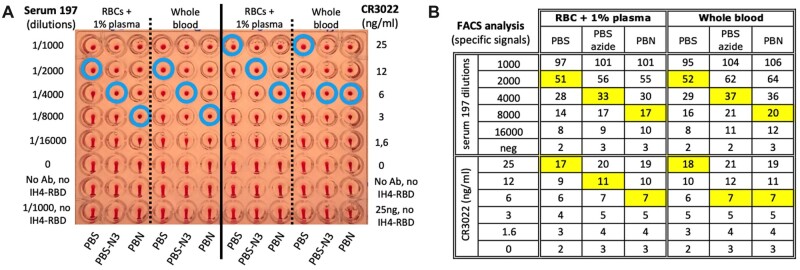
Azide and BSA increase the sensitivity of HAT. (**A**) The effects of azide and BSA on HAT performed using IH4-RBD at 1 µg/ml final concentration, and RBCs from a seronegative donor resuspended in PBS, PBS-N3 or PBN and 1% plasma from the same donor, or with whole blood from the same donor and with serial dilutions of an immune serum from a convalescent Covid-19 patient (Serum 197; left columns) or a monoclonal anti-RBD (CR3022; right columns). The three negative controls were: no antibody (0), neither antibody nor IH4-RBD and the most concentrated serum or antibody condition with no IH4-RBD. Blue circles indicate the titration endpoints (see the ‘Methods’ section for details). (**B)** After HAT, the RBCs were resuspended, stained with a fluorescent secondary anti-human Ig antibody and analyzed by FACS. The numbers shown correspond to specific signals, that is, the difference in GMFI values of each sample with that of the control sample incubated in the same buffer with no antibody or IH4-RBD. The squares highlighted in yellow indicate the titration endpoints. Comparable results were obtained in four similar experiments. (A color version of this figure appears in the online version of this article).

To investigate whether the presence of BSA and azide diminishes the performance of the HAT, we diluted the IH4-RBD reagent in PBS, in PBS containing 3 mM sodium azide (PBS-N3), or in PBN and used these diluted reagents to test haemagglutination of whole blood and O-RBCs from a seronegative donor resuspended in 1% plasma from the same donor, in the presence of serial dilutions of either the monoclonal anti-RBD CR3022 or of an immune serum (Fig. 2).

Rather than diminishing the performance of HAT, we found that the presence of azide improved its sensitivity: when HAT was performed in PBS-N3 rather than PBS, the titration endpoints (Fig. 2A, blue circles) were shifted by one double dilution (DD), and this occurred both with immune sera and with monoclonal antibodies. Addition of 1% BSA sometimes improved sensitivity by another DD, but we only saw this in some experiments, and not others.

To investigate the possible cause of the increased sensitivity of HAT in the presence of sodium azide, we used flow cytometry (FACS) to analyze the amount of antibody bound to the RBCs at the end of the HAT. Dilution of the IH4-RBD reagent in PBS-N3 or in PBN resulted in a small increase in the amount of antibody bound to the surface of the RBCs, but not to an extent that would explain the increased sensitivity (Fig. 2B). We postulate that the increased sensitivity due to the presence of azide may, instead, be primarily due to an ‘ageing’ effect on the RBCs.

### IH4-RBD is very stable when diluted in PBN

**Figure 3:**
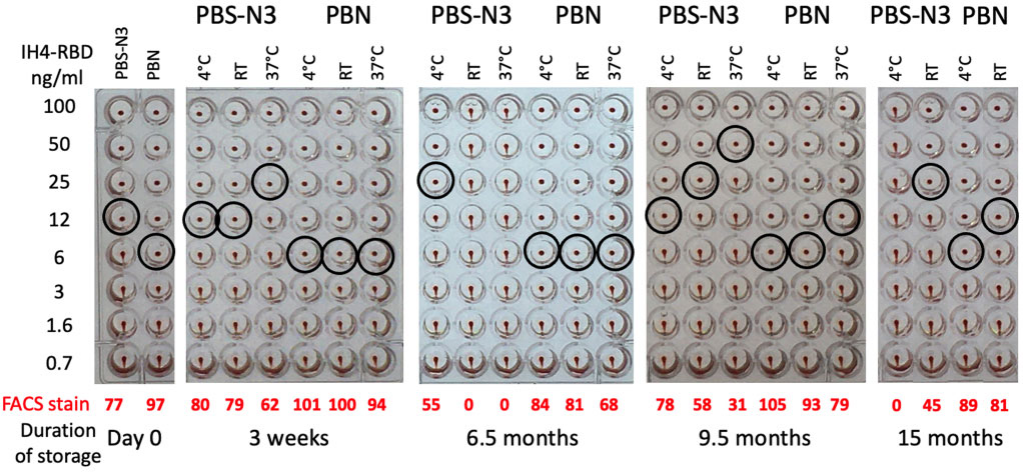
IH4-RBD is very stable when diluted in PBN. The effects of long-term storage of IH4-RBD in PBS-N3 or PBN at 4°C, RT and 37°C, as determined by HAT and by FACS analysis of antibody binding to RBCs after HAT. Haemagglutination end-points in the presence of the CR3022 monoclonal antibody at 100 ng/ml (black circles) were determined by titration of aliquots of working dilutions of IH4-RBD (2 µg/ml) stored for up to 15 months at the indicated temperatures. The red numbers indicate the intensity of the specific fluorescent staining recorded by FACS analysis performed after the HAT assay on the RBCs from the samples incubated with 100 ng/ml IH4-RBD (see the ‘Methods’ section for details). Similar data were obtained in seven experiments, some of which also included using diluted sera from convalescent patients in parallel to the CR3022 monoclonal antibody (not shown). (A color version of this figure appears in the online version of this article).

For use in the field, it would be most convenient for the IH4-RBD reagent to be pre-distributed in the wells, raising the question of the stability of working dilutions of the reagent, both in the cold and at ambient temperatures. To investigate the stability of IH4-RBD, we prepared aliquots of IH4-RBD at 2 µg/ml in either PBS-N3 or in PBN, on various dates over the course of 15 months and stored those aliquots at 4°C, room temperature (RT) or 37°C. Those IH4-RBD aliquots of various ages were then used to perform HAT titrations of the reagent in the presence of constant amounts of the CR3022 monoclonal antibody (Fig. 3). In some experiments, to evaluate more precisely the remaining activity of IH4-RBD after incubation, we also quantified the amounts of antibodies bound to the surface of the RBCs by using FACS (red numbers in Fig. 3).

By performing such experiments repeatedly, we found that IH4-RBD is remarkably stable when diluted in PBN: no significant loss of activity was seen for the IH4-RBD PBN dilution kept for 15 months at 4°C, and this was true for up to 6.5 months at RT. At 37°C, we did see some progressive loss of activity, but this only resulted in the loss of one DD in HAT sensitivity at 9.5 months (after 15 months, evaporation had caused the loss of what was left of the aliquots kept at 37°C).

On the other hand, the activity of IH4-RBD dilutions prepared in PBS-azide was usually already lower by one DD than those prepared in PBN on day 0. Furthermore, we observed marked variability over time between the IH4-RBD dilutions prepared in PBS-azide on different dates: some batches showed a drop of just one DD compared with the dilutions prepared in PBN, and stayed stable for many weeks after this; for others, however, we witnessed much more marked losses over time, dropping to undetectable levels after just a few weeks, even for tubes kept at 4°C. In retrospect, we suspect that this variability may be linked to the fact that, because the Covid-19 crisis had caused a penury of plasticware, different types and brands of plastic tubes had to be used to prepare and stock the IH4-RBD dilutions on different dates, and those different tubes probably had different protein-binding capacities, resulting in the variable loss of the diluted IH4-RBD protein.

The important take-home message we draw from this set of experiments is that, regardless of the brand or type of plastic tubes used, as long as IH4-RBD was diluted in PBN, the activity of the diluted stocks was always remarkably reproducible, and stable for over a year if kept at 4°C, and with only marginal losses for dilutions kept at RT or 37°C. This remarkable stability of IH4-RBD, which is the sole reagent required for HAT, could greatly facilitate making this serological test available to populations living in remote environments, with no access to refrigeration.

### Temperature has little influence on HAT results

**Figure 4:**
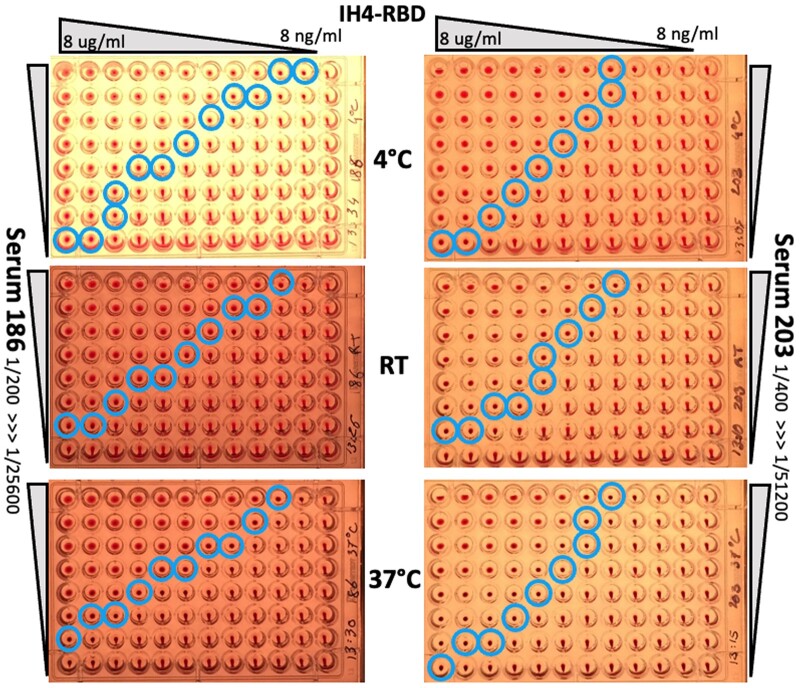
Temperature has little influence on HAT results. To determine the effect of temperature on the performance of HAT, three parallel plates were setup for 2D titrations, with DD of IH4-RBD going from 8 µg/ml to 8 ng/ml along lines and DD of two different immune sera from convalescent Covid-19 patients down columns (sera 186 and 203, see the ‘Methods’ section for practical details). After incubation at the indicated three temperatures, no substantial differences were seen in titration end-points (blue circles). Similar results were obtained in three independent experiments, using a total of three different immune sera, two plasmas and the CR3022 monoclonal antibody. Incubation temperature had little or no discernable influence on the haemagglutination endpoints (blue circles), with the possible exception of the wells containing the highest concentration of IH4-RBD and very diluted sera, where incubation at 4°C resulted in a small improvement in sensitivity when compared with the assays performed at RT or 37°C. (A color version of this figure appears in the online version of this article).

For use as a PoC test in the field, the HAT should be robust in many environments and, in particular, at a broad range of temperatures. To evaluate how temperature influences the results of the HAT, we set-up three identical 96-wells plates for 2D titration experiments (i.e. DDs of antibodies in one direction and of the IH4-RBD reagent in the other) and incubated those at three different temperatures: at 4°C (on ice in a cold room), at RT (ca. 21°C) and at 37°C (in a CO_2_ cell culture incubator; Fig. 4).

### The HAT-field protocol

**Figure 5:**
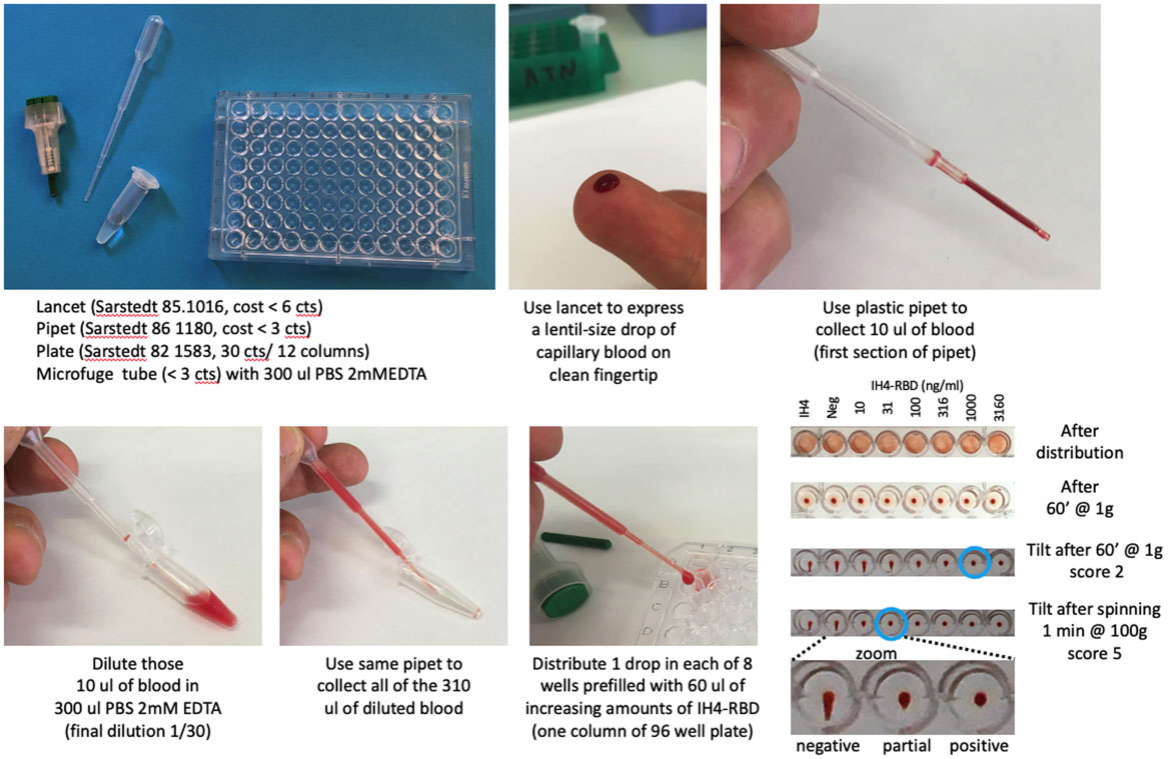
Schematic description of the HAT-field protocol.

The observation that, in 2D titrations such as those shown in Fig. 4, the titration endpoints were distributed in an almost linear fashion on an *X*–*Y* axis suggested to us that a quantitative version of HAT might be developed by using dilutions of IH4-RBD rather than by using serial dilutions of plasma or sera and donor RBCs. We have now devised such a quantitative protocol, which only requires, for each test, one lancet, one plastic Pasteur pipet, one plastic tube containing 300 µl of PBS—2 mM EDTA, 10 µl of whole blood, and one column of eight conical wells on a 96-well plate, preloaded with 60 µl/well of a range of concentrations of IH4-RBD (Fig. 5).

The lancet is used to express a lentil-size drop of capillary blood from a clean fingertip of the subject to be tested. The plastic Pasteur pipet is used to collect 10 µl of that blood, which corresponds to filling the first section of the pipet (the precise volume of blood collected is not critical; it may vary by as much as 30% with no detectable influence on the results). The blood is diluted, ca. 30-fold in the tube containing 300 µl of PBS—2 mM EDTA. The same pipet is then used to collect all 310 µl of this diluted blood and to transfer one drop into each of the eight wells of a 96-well plate, prefilled with 60 µl of PBN containing seven concentrations of IH4-RBD and a negative control well containing either PBN or IH4 alone (not fused to RBD) diluted in PBN to a similar molar concentration as the highest IH4-RBD concentration used. As for the original HAT, the plate is incubated at RT, tilted after 60 min and photographed after ca. 20 s. The photograph will later be used to score the samples. Scoring simply corresponds to the number of fully haemagglutinated wells in a column, and thus goes from 0 (no haemagglutination in the well with the highest concentration of IH4-RBD, i.e. 3.16 µg/ml) to 7 (full haemagglutination in the well with the lowest concentration of IH4-RBD, i.e. 3.16 ng/ml). For the tilting and the photographing, we find it convenient to use a very simple home-made light box (see tutorial) and a standard smart phone camera.

One limitation of HAT is that it is not as sensitive as ELISA, chemiluminescence immunoassay (CLIA) or FACS [8, 12]. As we have seen above (Fig. 4), increased sensitivity can be attained by the use of more IH4-RBD reagent, but we found that another way to increase HAT sensitivity was to perform prolonged incubations. After 5 h, for example, we saw a very significant improvement in sensitivity, with the titration endpoints increasing for most samples by 2 or 3 dilution points when compared with those after 60 min, with fewer and fewer samples that were detected positively by FACS remaining below the threshold value of 1 for HAT-field (Supplementary Fig. S1, first line).

Such long incubations are, however, not practical for a test intended for use in field settings. To overcome this problem, we found that centrifugation of the plates at 100 g for 1 min increased sensitivity to a level equivalent, or even slightly superior to that of incubating the plates for 5 h. This centrifugation step, moreover, may be performed 15 min after distributing the diluted blood in the plate, with similar results to those obtained if the plates were centrifuged after 60 min incubation (Supplementary Fig. S1, second line). With access to the means to centrifuge the assay wells (which can be achieved in adapted salad-spinners, see the ‘Discussion’ section), the HAT-field protocol can thus be completed in less than 30 min, which would be compatible with performing it in certain field settings, for example, in the context of vaccination centres, to identify individuals with high levels of antibodies, who might not need to be vaccinated or re-vaccinated.

### Validation of the HAT-field protocol

To validate the performance of the HAT-field protocol, we used a panel of 60 EDTA whole-blood samples collected in early September from patients in the hematology department of Toulouse University hospital. The samples were picked randomly from clinical samples left over after the prescribed hematology analyses had been performed. At that time, over 85% of the adult population had been vaccinated in France, and we thus expected a large proportion of the samples to be seropositive against the S protein of SARS-CoV-2, albeit at various levels.

**Figure 6:**
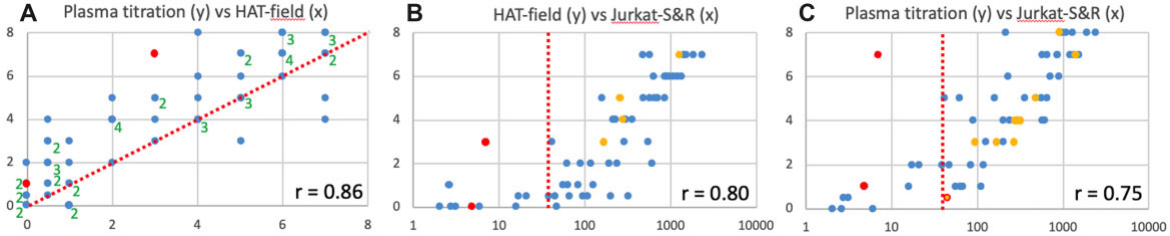
Validation of the HAT-field protocol by comparison with laboratory tests. Sixty randomly selected blood samples were used to compare the results of the HAT-field protocol with those of HAT plasma titrations and the Jurkat-S&R-flow test (see the ‘Methods’ section). The graphs show one-on-one comparisons of the results obtained with those three tests, as indicated, using the values obtained by centrifuging the plates after 15 min (for HAT-field), and after 60 min for plasma titrations. Pearson’s correlation coefficients are indicated in the bottom right corners. On panel A, because of the discrete nature of the scales used for both the *X-* and *Y*-axes, many points overlap on top of one another. In such cases, their numbers are indicated by the adjacent green numbers. The dotted line in A indicates the position of the median and those in B and C indicate the threshold for positive samples in the Jurkat-S&R-flow. The two red dots in each graph correspond to two negative samples, which gave false-positive results in HAT plasma titrations due to their reactivity against the IH4 nanobody moiety of the reagent. The orange dots correspond to samples positive in the Jurkat-S&R-flow test that showed some reactivity against the IH4 nanobody alone, albeit with lower titers than against the IH4-RBD reagent. For the sample represented by an orange dot surrounded by a red circle in C, the plasma titration against the nanobody alone was positive, but it led to only partial haemagglutination with the IH4-RBD reagent (for actual values, see sample 48 in data file). (A color version of this figure appears in the online version of this article).

The results obtained by HAT-field on these 60 whole blood samples were compared with those obtained by testing the plasma from the same samples by using the original HAT protocol with donor RBCs [4] and by using the FACS-based Jurkat-S&R-flow test [12], which is very sensitive, quantitative, and allows isotyping of the antibodies reacting against the S protein (Fig. 6).

The very good correlation between the results of all three tests validates that the HAT-field protocol can be used for quantitative assessment of the levels of antibodies contained in a whole-blood sample in a single step, without the sophisticated equipment needed for the Jurkat-S&R-flow test and without needing to separate the RBCs from plasma or serum and having access to RBCs from an O-donor, as in the experiment using the original HAT to perform plasma titrations.

### HAT-field works with Delta-variant IH4-RBD

Our IH4-RBD reagent was designed to present the RBD sequence of the original Wuhan variant (residues 340–538 of the S protein [4]). At the time of our study, however, a large proportion of the SARS-CoV-2 viruses circulating in France belonged to the Delta variant lineage (see epidemiological report here https://www.santepubliquefrance.fr/maladies-et-traumatismes/maladies-et-infections-respiratoires/infection-a-coronavirus/documents/bulletin-national/covid-19-point-epidemiologique-du-30-septembre-2021), which has two mutations in the RBD domain (L452R and T478K in the B.1.617.2 strain) [6, 13]. We therefore wanted to compare the results obtained with the IH4-RBD-Wuhan reagent (used above) with a reagent that incorporates the two mutations in the Delta variant, IH4-RBD-Delta. We tested the 60 blood samples with the IH4-RBD-Wuhan and IH4-RBD-Delta reagents in both the HAT-field and original HAT plasma titration assays. For most samples, the scores obtained were one or two units higher when the Wuhan IH4-RBD reagent was used than those with the IH4-RBD-Delta reagent (Supplementary Fig. S3). This is consistent with a previous report that, in vaccinated people, HAT titers obtained with the IH4-RBD-Delta tend to be lower than with the IH4-RBD-Wuhan [13], and with the fact that, at the time of our study, most people in the French population had antibodies due to being vaccinated and not as a consequence of a previous infection by the SARS-CoV2 virus [retrospective analysis of clinical information on our cohort of 60 blood samples revealed that only three samples were from patients who had ever had a positive PCR test for SARS-CoV-2 (see data file and Supplementary Fig. S3)].

## Discussion

In this article, we describe an adaptation of the HAT protocol which is quantitative, shows satisfactory sensitivity and can be used in the field with no specialized laboratory equipment such as adjustable pipets and disposable tips. This was made possible by the observations that (i) the use of PBN results in markedly improved HAT robustness and sensitivity, (ii) HAT sensitivity is markedly improved by prolonged incubations (or by brief low-speed centrifugation), albeit with a parallel drop in specificity and (iii) quantification could be achieved by titrating the IH4-RBD reagent rather than the plasmas or sera. Incidentally, we realized recently that such an approach of titrating the RBC-binding reagent had been suggested previously for an HIV serodiagnostic test [14].

PBN is a buffer containing both 1% BSA and azide, which has several concomitant advantages: (i) Used as a dilution buffer for the IH4-RBD, it blocks the reagent’s nonspecific adsorption to plastic and results in its much improved stability over time, even if kept out of the cold. (ii) The conjoint action of azide and BSA results in slightly increased sensitivity, probably because they both improve the settling of the RBCs at the bottom of the wells. (iii) In experiments which involve the use of O-RBCs, for example, when using HAT to titrate plasmas or sera, the use of whole blood (or the addition of 1% seronegative plasma or serum) is no longer necessary when PBN is used to prepare a suspension of washed O-RBCs. We feel that this advantage is quite significant since most blood donors in the population have now become seropositive because of vaccinations.

Once we had found that the use of centrifugation, combined to that of PBN as a buffer, could result in a marked improvement of the sensitivity of the ‘standard’ HAT assay in test samples, we needed to validate the performance of the modified HAT assay on clinical samples. For this, we made use of a cohort of 60 clinical blood samples of unknown serological status, and characterized those using the Jurkat-S&R-flow test, which is both extremely sensitive, quantitative and allows the semi-quantitative isotyping of the plasmatic antibodies [12]. As seen in Fig. 6, we found very good correlations between all three tests: HAT-field, HAT plasma titrations and the Jurkat-S&R-flow test.

While HAT was, from its initial conception, always intended primarily to be carried out on capillary blood obtained by fingertip pricks, because of regulatory restrictions, all the experiments described in this article had to be performed on samples of venous blood collected by phlebotomy. A recent study has shown that, as could be expected, HAT performed on capillary blood gives the same results as on venous blood samples [6], and preliminary results which we have obtained recently suggest that the results of the HAT-field protocol performed on capillary blood indeed correlate just as well with those of the Jurkat-S&R-flow test as those obtained with venous blood (E. Joly et al., manuscript in preparation).

On the graph on the left of Fig. 6, which compares the scores of titrations by standard HAT to those obtained with HAT-field, most points are sitting above the median line, with 11 points not scoring positive by HAT-field (i.e. a score <1) while being positive by standard HAT titration (i.e. score ≥1). In the conditions used in this study, the HAT-field protocol was thus markedly less sensitive than the optimized standard HAT approach, which can be explained by the conjunction of four factors:

- For titrations by standard HAT, we used plasmas at 1/50 as the highest concentration. In other words, we used 2 µl of plasma per well, to react against 0.3 µl of RBCs. The plasma-to-RBC ratio was thus six times more than in HAT-field, where there are roughly equivalent volumes of RBCs and plasma, and this ratio of 1 cannot be altered since, in HAT-field, the blood samples are simply diluted before performing the assay. On this subject, we have found that increasing the amount of whole blood per well (in other words, using blood that is less dilute) has very little influence over the HAT-field results, and, if anything, adding more blood can sometimes reduce the sensitivity, albeit never by more than 1 dilution.
- Titrations for Fig. 6 were carried out on washed RBCs from an O-donor that were 5-days old, and, in our hands, RBCs which have been washed and stored at 4°C for a few days tend to work a bit better for HAT than those in whole blood.
- When using washed RBCs, EDTA is no longer present and we have found that excess EDTA can reduce HAT sensitivity for certain samples, possibly because the binding of certain antibodies may involve divalent cations. In the future, it may thus be interesting to explore the possibility of using heparin rather than EDTA as an anticoagulant.
- While plasma titrations were carried out by DDs of the plasmas, in the optimized protocol we have devised for HAT-field, the IH4-RBD reagent is titrated in steps of 3.16-fold, so as to cover a broader range (see the ‘Methods’ section). This does, however, only really influence the upper right corner of the graph, that is, the scores of samples with very high levels of antibodies.

On Fig. 6C, one finds four samples that scored positive by standard HAT, while the staining values in the Jurkat-S&R-flow test were in the doubtful zone between 10 and 40, and our view is that those samples probably contained some antibodies reacting specifically against the SARS-CoV-2 spike protein, and their more effective detection by haemagglutination than by FACS staining may be due to relatively high proportions of IgAs or IgMs. This is indeed reminiscent of the observation reported in our first paper that, during very early SARS-CoV-2 infections, HAT could detect antibody responses before CLIA [4]. Incidentally, although this is not something that we have yet managed to document formally, we noticed that the samples which contain sizeable amounts of IgMs and/or IgAs often reach higher HAT scores than expected from the FACS results obtained with the pan-human Ig secondary antibody, with even higher scores by plasma dilutions than in HAT-field (see data file). Of note, for sample 19, which was a false positive and gave very high titers against that IH4 alone, FACS analysis performed after the HAT assay showed that the human antibodies bound to the IH4-coated RBCs were predominantly IgMs (data not shown).

For one sample (n° 48 in data file), indicated by an orange symbol with a red circle in Fig. 6C, the result of Jurkat-S&R-flow test was 44.84, that is, just above the threshold value which is arbitrarily set to 40. This sample only led to partial haemagglutination at 1/50 in the standard HAT, but showed reactivity against the IH4 nanobody alone with an endpoint at 1/200 after centrifugation (score 3). We thus surmise that this sample contained either very low or no specific anti-SARS-CoV-2 antibodies, which provides a justification for maintaining the Jurkat-S&R-flow test threshold at its current value.

As in all biological tests, increasing the sensitivity will almost unavoidably lead to an increase in the proportion of false positives. It is thus not surprising that, with the gain of sensitivity of HAT afforded by the combined use of PBN and spinning, the proportion of samples being detected as showing some reactivity against the IH4 nanobody should be higher than the 1–2% that were originally detected with the standard HAT protocol in various cohorts [4, 12].

As can be seen on the table of data for the cohort which is provided as the Supplementary Material, with the HAT-field protocol, after 1 h under normal gravity, reactivity against the IH4 moiety was detected in 3% of samples (2 out of 60), and climbed to 8% after spinning (5 out of 60). With the more sensitive protocol used for plasma titrations, five samples (8%) reacted against the IH4 nanobody after 1 h under simple gravity, but this number climbed to 12 (20% of samples) after spinning. If the HAT-field test was ever to be used in a clinical context, the results would be invalidated for those samples found to react with the IH4 alone, and with such high frequencies as we have observed in our small cohort, this could be a significant problem. An alternative would be to perform, from the start, systematic parallel titrations of the IH4-BRD and IH4 alone reagent, in order to identify samples which react markedly better on IH4-RBD than on IH4 alone. This could be achieved either by using 20 µl of blood diluted into 600 µl to distribute in two sets of eight wells, which would be quite easy since 20 µl corresponds to the second section on the plastic Pasteur pipets. Alternatively, titrations of the two reagents could be carried out over just four wells each, with larger dilution factors between wells (e.g. 10-fold).

In future, to reduce the proportion of false positives due to reactivities with the IH4 moiety, it may be interesting to investigate if the nanobody, which is of camel origin, could be somewhat ‘humanized’ by site-directed mutagenesis without losing its capacity to bind to human glycophorin. Alternatively, the use of different antibodies binding to glycophorin, such as nanobodies derived from other species, or mAbs such as the one described by Kemp et al. [14, 15] could also be explored as an alternative.

Two recent reports have described that HAT could be performed on cards rather than in V-shaped wells, with semi-quantitative results being obtained in minutes, which was made possible by the use of much higher amounts of the IH4-RBD reagent than when HAT is performed in V-shaped well [16, 17]. Reliably quantitative card-based tests HAT test would unquestionably be a very attractive solution for performing the test in field settings. Comparing the sensitivity, specificity and robustness of the two types of protocols, on cards or in V-shaped wells, on the very same cohorts of whole blood samples would be very interesting, especially if such comparisons were performed by third-party laboratories.

## Conclusion

We have shown that the HAT-field approach, which uses a single drop of capillary blood, can provide, in a single step, a quantitative measurement of the antibodies against the viral RBD, which are those endowed with neutralizing activity. Such a test could prove very useful for identifying individuals in need of a vaccine boost (or a primary injection). Given its very low cost, the stability of the IH4-RBD reagent, and the simplicity of the procedure, HAT-field should be well suited to be performed pretty much anywhere, including in the poorest countries and the most remote corners of the globe. Given that HAT has already been successfully adapted to detect antibodies against the RBD of several SARS-CoV-2 variants [6, 7], we presume that it could be adapted very rapidly to evaluate the levels of antibodies reacting against the RBD of other newly arising SARS-CoV-2 variants of concern, such as the newly arisen and very divergent Omicron variant, or, in future, to pretty much any new threatening pathogen, and could thus represent a great asset to be better prepared to face future pandemics.

## Methods

### Reagents

PBS and tissue culture media were all obtained from Gibco.

BSA Fraction V was obtained from Sigma (ref A8022 or A7888). Of note, we have tried using other sources of Fraction V BSA for preparing PBN and found that they do not all work as well as the ones listed above to prevent veil formation in HAT.

Sodium azide (NaN3, Sigma S2002) was prepared as a 20% stock solution in milli-Q water and kept at RT. This 3M solution was then used as a 1000× stock for the preparation of PBN, PFN and PBS-azide.

PBN was prepared by adding 500 µl of the above 1000× azide stock and 5 g of BSA Fraction V (Sigma A8022) to 500 ml of PBS.

PFN, used for the dilution of antibodies and washes of FACS samples, was prepared by adding 500 µl of the above 1000× azide stock and 10 ml of fetal calf serum to 500 ml of PBS (we find that this is a very good use for unwanted or expired stocks of FCS that often clutter the bottom of freezers).

Because they contain azide (final concentration 3 mM), no sterilizing filtration is needed for either PBN or PFN, and they can be kept for many weeks at 4°C.

Polyclonal anti-human Igs secondary antibodies, all conjugated to Alexa-488, were from Jackson laboratories and purchased from Ozyme (France). Refs: anti-human Ig-GAM: 109-545-064, Ig-G: 109-545-003, Ig-A: 109-54-011 and Ig-M: 109-545-129.

Anti-RBD monoclonal antibodies: CR3022 [18] and EY6A [19] were obtained using antibody-expression plasmids, as previously described [4].

Covid-19 sera 186, 197 and 203 were obtained from the virology department of the Toulouse hospital; plasmas 79 and 206 were from whole blood samples used in our previous paper describing the Jurkat-S&R-flow test [12].

IH4-RBD (Wuhan and Delta) and IH4 alone were produced by transient transfection of HEK-293T cells, and purified from the supernatant by HIS-tag affinity purification, as previously described [4]. Samples of IH4-RBD, sufficient for several thousand tests, or plasmid encoding IH4-RBD can be obtained by following the request procedure explained in that first manuscript. Of note, IH4-RBD reagents carrying the RBDs corresponding to several SARS-CoV2 variants have also been generated. For the experiments described here, highly concentrated stock solutions at 3–5 mg/ml in PBS were kept frozen as aliquots. Those were then used to prepare 100× stocks in either PBS or PBN, which were kept frozen, and kept a 4°C after thawing for up to a few weeks.

To study the stability of the IH4-RBD reagent over time, working solutions at 2 µg/ml in either PBS-azide or in PBN were prepared from 100× PBS stocks at various time points over the course of a whole year. Three aliquots of 500 µl each were then set aside from those working stocks, to be kept either at 4°C, at RT or at 37°C in a tissue culture incubator. The activity of those aliquots of working stocks was then evaluated at regular intervals by performing titrations of the IH4-RBD reagent against either the CR3022 monoclonal antibody diluted to a final concentration of 100 ng/ml, or various immune sera diluted to give similar endpoint to those obtained with CR3022.

When comparing the reactivities against the Wuhan and Delta IH4-RBD reagents, we ascertained that we were using working concentrations of the two reagents with comparable activities by performing titrations with two monoclonals, EY6A or CR3022, which recognize a binding site not affected by the two mutations carried by the RBD of the Delta viral lineage, and found the Wuhan and Delta reagents to have indistinguishable haemagglutinating activities under those conditions.

### Ethical statement

RBCs from O-blood donors were obtained from the Toulouse branch of the Etablissement Français du Sang (EFS), with whom the project was validated under agreement n° 21PLER2020-025.

Whole blood samples: The 60 samples used for Fig. 6 and Supplementary Figs S1–S3 were routine care residues from patients of the Toulouse hospital, where all patients give, by default, their consent for any biological material left over to be used for research purposes after all the clinical tests requested by doctors have been duly completed.

According to the French law on ethics (loi Jardé), retrospective/prospective studies based on the exploitation of clinical care residues do not require to be submitted to an ethics committee. This study was reviewed and approved by the ‘Directeur de la Recherche et de l’Innovation du CHU de Toulouse’, who confirmed that this study conformed with all the ethical and legal requirements, and gave his signed approval with the agreement number RnIPH 2021-99. This was further confirmed by the signature of a Material Transfer Agreement between the Toulouse University Hospital and the CNRS, with the following references: Toulouse Hospital n° 20 427 C and CNRS n° 227232.

### Human samples

The 60 whole blood samples were collected, regardless of gender, in the course of the month September 2021. Those were anonymized within 24 h of collection, transferred from the hospital to the research lab and kept at RT until being used for HAT assays within 24 h (i.e. less than 48 h after blood samples were collected). In trial experiments, we had found that such samples could be stored for up to 5 days without any noticeable difference in the performance of the HAT tests.

After the whole blood samples had been used for HAT-field assays, the tubes were then spun, the plasmas harvested into fresh tubes and sodium azide added to 3 mM final. Those harvested plasmas were kept at 4°C until they were used to perform the Jurkat-S&R-flow tests and HAT assays for plasma titrations.

The identities, clinical conditions and Covid status (PCR or positive serology) were unknown to the person performing the HAT experiments and Jurkat-S&R-flow tests.

Blood samples from O-donors (6 ml EDTA tubes) were obtained every few weeks from the EFS (Toulouse blood bank). While whole blood is best kept at RT (i.e. between 20°C and 25°C), and can then be used for HAT assays for up to 5 or 6 days, washed red blood cells can be kept for several weeks, as long as they have been separated from the white blood cells and are kept at 4°C in the right buffer (in our case Alsever’s solution, Sigma A3551).

For preparing RBCs for storage at 4°C, we used the following standard protocol. The EDTA collection tube is spun at 1000 g for 20 min with no refrigeration, and the centrifuge brake set to 2/9. The plasma is collected into a separate sterile tube and a total of 8 ml of sterile PBS used to resuspend the cells and transfer them to a 15-ml tube, on top of a 3-ml cushion of lymphocyte separation medium (Corning Ref 25–072-CV). This tube is then spun once more at 1000 g for 20 min with no refrigeration, and the centrifuge brake set to 2/9. The supernatant, including the ring of white blood cells, is aspirated and discarded. The RBC pellet is then washed twice with 8 ml sterile PBS, once in 8 ml Alsever’s solution, before adding two volumes of Alsever’s solution to the one volume of packed RBCs. This tube of RBCs (at 30% v/v) can then be stored at 4°C and used for HAT assays for several weeks. After a week of storage, however, we found that the RBCs progressively tend to lose their capacity to teardrop after spinning.

If whole blood from an O-donor was needed for experiments (e.g. as in Fig. 2), 100–200 µl were transferred to a separate sterile tube before performing the above procedure and this tube was kept at RT for a maximum of 4 days. Worthy of note, while all donors were seronegative at the start of this study (i.e. from summer 2020 to winter 2021), the proportion of seropositives started increasing in the spring 2021, correlating with the proportion of vaccinated people increasing in the French population, and we have come across no seronegative samples among the dozen of blood samples we have used since the beginning of the summer 2021. While the serological status of the donors does not matter when working with washed RBCs, it would thus no longer be practical to plan performing HAT tests in the presence of 1% autologous plasma as originally recommended [4], but, as shown in Fig. 1, this is no longer necessary in the presence of 1% BSA.

### Original HAT assays

For HAT assays performed under ‘standard’ original HAT conditions (Figs 1–4), outside of using PBN instead of PBS, we used similar reagent concentrations and incubation conditions to those defined in our original description of HAT [4]:

- in a final volume of 100 µl per well;
- with approximately 0.3 µl of packed RBCs per well, that is, 1 µl of 30% stock stored in Alsever’s solution;
- using IH4-RBD at a final concentration of 1 µg/ml (i.e. 100 ng/well), or alternatively 0.5 µg/ml of IH4 alone since the size of the nanobody and the His tag corresponds to slightly less than half of that of the IH4-RBD recombinant protein;
- Taking pictures after incubating the plates for 60 min at RT, for which we find it very convenient to use a very simple home-made lightbox (https://youtu.be/e5zBYd19nIA).

For experiments such as that presented in Fig. 1, tubes of the appropriate 2× stocks containing either the RBCs and the antibodies or the IH4-RBD reagent were prepared to be mixed 50/50 in each well.

For titration experiments, we prepared stocks containing RBCs and the appropriate amount of the reagent to be kept constant (either the IH4-RBD reagent at 1 µg/ml or IH4 alone at 0.5 µg/ml for plasma titrations or the appropriate antibody dilution for IH4-RBD titrations). In total, 100 µl of those stocks were then distributed per well and 200 µl for the wells of the first row. The reagents to be titrated were then added to each of the wells of the first row, and a multi-channel pipet was then used to perform the serial dilutions by successive transfer of 100 µl to the wells of the adjacent row, with thorough mixing by pipetting gently up and down at least six times at each diluting step.

A similar method was used to perform the 2D titrations presented in Fig. 4, filling columns of wells with stocks of IH4-RBD serially diluted with the appropriate suspension of RBCs, and proceeding in a second stage to perform serial dilutions of the antibodies in rows, as described above.

### HAT-field assays

The optimized procedure we have arrived at and used for this study is based on using seven serial dilutions of the IH4-RBD reagent. Rather than DDs, we elected to use 3.16 as a dilution factor between adjacent wells. This not only allows to cover a larger range of IH4-RBD concentrations, but because 3.16 is the square root of 10, two successive dilutions conveniently correspond to a factor of 10. The concentrations of the IH4-RBD stocks used to prefill the wells were thus, in ng/ml 4750, 1500, 475, 150, 47, 15 and 4.7. After addition of one drop of diluted blood, that is, roughly 30 µl, the final volume was thus ca. 90 µl and the approximate final concentrations of IH4-RBD in the wells were thus, in ng/ml: 3160, 1000, 316, 100, 31, 10 and 3.1.

A detailed step by step protocol on how to generate the stocks of those various dilutions, and how to perform the HAT-field test, is provided as the Supplementary Material.

For every sample, the eighth well of a row is allocated to performing the very important negative control, which can consist of either PBN, or preferably PBN containing the IH4 alone, that is, the nanobody without the RBD attached to it, at 1.6 µg/ml final concentration, corresponding to a molar concentration similar to that found in the well with the highest concentration of IH4-RBD.

For the 60 samples of the cohort used in our study, because we were running sets of parallel HAT-field assays with either the IH4-RBD-Wuhan or IH4-RBD-Delta reagents, we performed both types of negative controls by using PBN as a negative control in the plates for the Wuhan HAT-field assays, and IH4 alone as a negative control in the plates for the Delta HAT-field assays. As discussed above, we found that the negative controls using the IH4 alone were much more informative.

For spinning the plates at 100 g for 1 min, we used a centrifuge with swinging-out trays (Eppendorf 5810R, set to 705 rpm).

### Scoring HAT

The procedure we used for scoring HAT tests was based on the same principle for plasma titrations using the original HAT protocol and for HAT-field: At the end of the incubation period, the plates were tilted to an almost vertical position using a purposefully designed homemade light box (see protocol provided and online video tutorial). The pictures used for scoring were taken with a mobile phone camera after about 20 s, that is, when the teardrops in the negative control wells reached the bottom walls of the wells.

After transfer of the pictures to a computer, scoring was then carried out by simply counting the number of fully positive wells, starting either from the highest IH4-RBD concentration for HAT-field, or the least diluted plasma for titrations if using the original HAT protocol.

**Figure 7:**
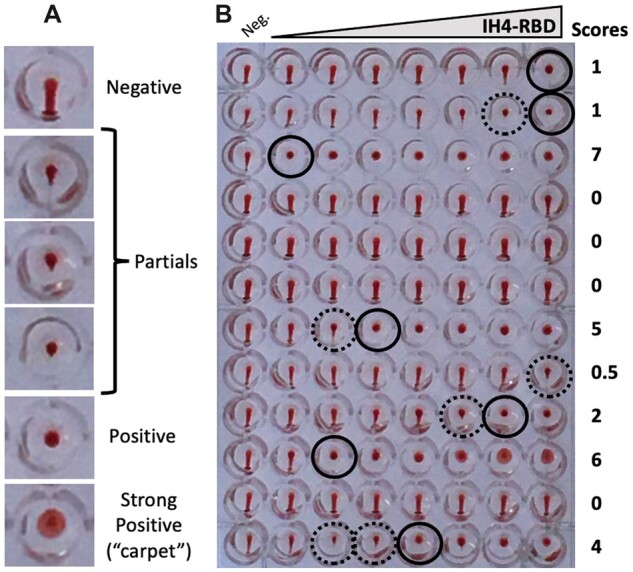
Scoring HAT. (**A**) Examples of typical negative, partial and positive wells. Wells are considered as partial as soon as a small point appears at the bottom of the RBD pellet, such as that shown on the fourth line. (**B**) Example of a plate with HAT-field performed with Wuhan IH4-RBD on 12 whole blood samples after 60 min under normal gravity. The final IH4-RBD concentrations in the wells from left to right are, respectively, 0, 3, 10, 31, 100, 316, 1000 and 3160 ng/ml. Black circles indicate the last positive wells, and dotted circles partially positive ones. Scoring is carried out on the pictures by simply counting the number of positive wells, starting from the highest IH4-RBD concentration (i.e. from right to left).

The trickiest part in scoring HAT lies with separating partially from fully positive wells. For this, we find that the most reliable means consists in considering any RBC pellet that shows a detectable pointy bottom as only partially positive (e.g. the fourth sample from the top in Fig. 7A).

With certain samples containing high amounts of antibodies, it is quite frequent to see the RBCs forming broader pellets, which we refer to as ‘carpets’ since those have a certain tendency to float off the bottom of the wells and fold upon themselves (see strong positive in Fig. 7A and sample on line 10 of Fig. 7B). Those carpets, which disappear upon centrifugation at 100 g, are quite different from the veils due to the adsorption of IH4-RBD to the wells’ bottom in the absence of BSA (Fig. 1A).

While the tendency of certain samples to form carpets is highly reproducible, it is not seen for all samples with high levels of antibodies. For example, the sample on line 3 of Fig. 7B had even higher levels of antibodies than that on line 10, as determined both by HAT-field, plasma titration and FACS, but did not form carpets. Of note, the Ig-G/A/M profiles of samples as determined by FACS showed no obvious correlation with their tendency to form those carpets.

### Practical considerations for performing HAT assays

When performing HAT in PBN compared with PBS, we observed a slight increase in sensitivity which we suspect is most probably due to an improved sedimentation of the RBCs, with both azide, and BSA, contributing to the formation of more compact pellets at the bottom of the V-shaped wells. Regarding the role of azide, we postulate that, by blocking the metabolism of the RBCs, it probably increases their density and consequently their sedimentation. Regarding the beneficial role of facilitating the sedimentation of RBCs, this is something that Wegmann and Smithies [20] had recognized soon after their initial description of the microtiter haemagglutination method, and for which they had proposed using paraffin-coated plates as an improvement.

As we have seen, the improvement of HAT sensitivity provided by prolonging incubations up to 5 h can be very advantageously replaced by a brief step of centrifugation at 100 g, which can be performed after only 15 min of incubation. The need for a centrifuge with the capacity to spin 96-well plates would, however, rather preclude the possibility of performing HAT in the field. But accelerations of 100 g are in the range of those attained by hand-driven centrifuges such as salad spinners. We have investigated the possibility of using this type of centrifuge for spinning 96-well plates after just 15–20 min of incubation, but have found that this only works for the central two columns of a 96-well plate because the RBCs in the outer columns are pushed to the outside of the wells. While it is hard to conceive that hand-driven centrifuges with the capacity to spin 96-well plates in swinging-out trays could become part of the equipment enabling the use of HAT in the field, a rather simple solution would be to design plastic strips of conical wells for individual tests. Using very simple adapters, those purposefully designed sets of wells could then be spun in hand-driven centrifuges of the salad spinner type. One aspect that will have to be considered for the design and use of such individual strips of wells will be to ensure that, upon storage, the various dilutions of IH4-RBD are as stable in such strips as the working stocks of IH4-RBD (2 µg/ml) tested in Fig. 3. The design, and manufacture, of such disposable strips of wells, which would necessarily require the involvement of an industrial partner, was, however, well beyond the means of this study.

One important consideration about performing centrifugation at the end of a HAT assay is that, while this works very well with RBCs contained in whole blood samples, or with washed RBCs from freshly collected blood, we found that it can become problematic with RBCs that have been stored at 4°C for more than a week. Over time, stored RBCs will indeed progressively lose their capacity to teardrop, which can, incidentally, result in a slight improvement of the apparent sensitivity of HAT assays performed under simple gravity.

But, if submitted to accelerations of 100 g, we have found that such ‘aged’ RBCs will form compact pellets that will fail to teardrop, even upon prolonged tilting of the plate (of note, this is also the case for RBCs that have been kept in azide for a few hours. Preparing suspensions of RBCs in PBN should thus be done just before performing the HAT assays).

In their original paper, Wegmann and Smithies [10] had described using incubations of 4–6 h as their standard protocol, and suggested that a brief step of centrifugation could be used as an alternative. For the record, during the initial stages of this study, we had made several attempts to use centrifugation, but we did not have access to whole blood samples at the time. We were thus using O-RBCs which had often been stored at 4°C for up to several weeks and had initially given up on centrifugation because the aged RBCs we were using failed to teardrop after centrifugation. Incidentally, in that same paper, Wegmann and Smithies [10] also suggested using BSA as an alternative to serum or plasma to promote better RBC settling patterns. They were, however, recommending using BSA at 0.22% which we found to be less effective than 1% to block the adsorption of the IH4-RBD reagent to plastic.

### Jurkat-S&R-flow test

Briefly, Jurkat-S and Jurkat-R cell lines, obtained and grown as previously described [12], were resuspended in their own tissue culture medium at a concentration of 2.2 10^6^ cells/ml before pooling equal volumes of the two.

Plasmas to be tested were diluted 1/10 in PFN (PBS/2% FCS/200 mg/L sodium azide). Twenty microliters of these 1/10 dilutions were then placed in U-bottom 96-well plates, before adding 180 µl per well of the Jurkat-S&R mix.

The plates were then incubated for 30 min at RT before placing them on ice for a further 30 min. All subsequent steps were carried out in the cold, with plates and washing buffers kept on ice. After the primary staining, samples were washed in PFN, with resuspending the cells by tapping the plate after each centrifugation, before adding 150 µl of PFN for the next wash. After two washes, the samples were split into four wells and all resulting samples were washed one last time.

One drop (i.e. ca. 30 µl) of either the pan-specific anti-Ig-GAM secondary fluorescent antibodies, as well as anti-IgG, anti-IgA or anti-IgM, all diluted 1/200 in PFN was added to each of the four wells for each sample and the cells resuspended by gentle shaking of the plates. After an incubation of 60 min on ice, samples were washed two more times with cold PFN before transferring the samples to acquisition tubes in a final volume of 300 µl PFN containing 30 nM TO-PRO™-3 Iodide (Thermo Fischer Scientific, ref T3605).

The samples were then analyzed on a FACScalibur flow cytometer controlled by the Cellquest pro software (Version 5.2, Beckton Dickinson), using the FL1 channel for Alexa-488, the FL3 channel for m-Cherry and the FL4 channel (with the 633-nm laser) for live gating with the TO-PRO™-3 live stain. Post-acquisition analysis of all the samples was performed using the Flowjo software (version 10.7.1). The values used as results are those for specific staining, that is, the difference between the geometric mean fluorescent index (GMFI) measured on the Jurkat cells expressing the SARS-CoV-2 spike protein and the control Jurkat cells expressing the mCherry fluorescent protein. The value of 40 of specific staining was used as the threshold above which the samples were considered as positive. As described previously, with the cytometer settings used in this study, this correspond to 20-fold the value obtained with cells stained just with the secondary antibody [12].

### FACS analysis of RBCs after HAT

To quantify the amount of antibodies bound to the RBCs’ surface after a HAT assay, an adjustable pipet was used to resuspend the RBCS by pipetting up and down several times, and 15 µl (out of 90 or 100) were transferred to the well of a U-bottom 96-well plate pre-filled with 150 µl PFN. The RBCs were then washed by three repeated sequences of centrifugation at 800 g for 3 min, followed by flicking the supernatant out, tapping the plate and adding 150 µl of PFN. One drop (i.e. ca. 30 µl) of anti-human secondary antibody conjugated to alexa-488, diluted 1/200 in PFN was added to each of the wells and the cells resuspended by gentle shaking of the plates. After an incubation of 60 min on ice, samples were washed two more times with cold PFN before transferring the samples to acquisition tubes in a final volume of 300 µl PFN. The samples were then analyzed on a FACScalibur flow cytometer controlled by the Cellquest pro software (Version 5.2, Beckton Dickinson). Post-acquisition analysis of all the samples was performed using the Flowjo software (version 10.7.1).

Because they can be obtained in very large numbers and stored at 4°C for several weeks, it is actually much simpler to use RBCs for FACS analysis than Jurkat cells, which need to be kept in culture continuously. But we find that FACS analysis of RBCs has a much reduced dynamic range compared with the Jurkat-S&R-flow test. It is indeed less sensitive, and many samples harboring low levels of antibodies would not be detected by RBC staining. And for samples that contain very high amounts of antibodies, the RBCs will tend to stay agglutinated and the resulting cell clumps will be discounted during FACS analyses, with samples containing much lower numbers of usable cells, and the FACS results skewed towards lower values. If wanting to perform FACS analysis of RBCs after HAT, a solution to avoid this problem of clumping is to analyse those samples that are just one or two dilutions above the agglutination endpoint. But this will mean that all samples will not all have been stained with the same amounts of reagent and consequently that the staining levels cannot be compared with one another.

Alternatively, the problem can also be avoided by keeping the concentrations of the IH4-RBD below 100 ng/ml, but this will result in a further reduction of the sensitivity for the samples with low levels of antibodies.

## Supplementary Material

bpac026_Supplementary_Data

## Data Availability

The data underlying this article are available in the article and in its online supplementary material.
