# Supplementary material for: HAT-field: a cheap, robust and quantitative Point-of-care serological test for Covid-19
Source: Biol Methods Protoc. 2022 Nov 28;7(1):bpac026. doi: 10.1093/biomethods/bpac026 (PMC9620368; doi:10.1093/biomethods/bpac026)

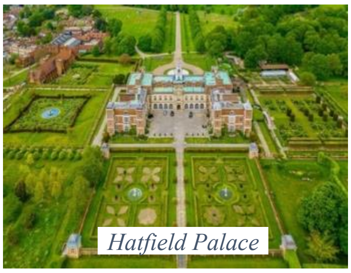

Protocol for HAT-field

(Etienne Joly, atnjoly@mac.com)

Equipment Required

Equipment for laboratory procedures

- Adjustable pipets + tips
- 15 ml tubes and 10 ml pipets
- Small tubes (e.g. Eppendorf tubes)
- V bottom 96 well plate (e.g. Sarstedt 82.1583)

Reagents

- **RBC dilution** = PBS + 2 mM EDTA
- **PBN** = PBS + 1% BSA + 200 mg/l azide
- **100 X Stock of IH4 alone** @ 240 µg/ml in PBN
- **100 X Stock of IH4-RBD** @ 475 µg/ml in PBN
- Control Antibody CR3022 @ 20 ng/ml in PBN (other mAbs, e.g. EY6A can also be used)

Equipment for HAT-field procedure

- Disposable lancets (e.g. Sarstedt 85.1016) , sterile swabs, alcohol
- Small tubes (e.g. Eppendorf tubes) containing 300 µl of RBC dilution (PBS + 2 mM EDTA)
- Plastic Pasteur pipettes (small volume e.g. Sarstedt 86.1180)
- V bottom 96 well plates, if possible prefilled with 60 µl/well of working solutions (see below).

Preparation of working solutions:

Rather than simple DD, we elected to use a factor of 3.16 between successive dilutions. This not only allows to cover a larger range of IH4-RBD concentrations, but because 3.16 is the square root of 10, two successive dilutions correspond to a factor of 10. The concentrations of the IH4-RBD stocks used to prefill the wells will thus be, in ng/ml 4750, 1500, 475, 150, 47, 15, 4.7. After addition of one drop of diluted blood, i.e. roughly 30 µl, the approximate final concentrations of IH4-RBD in the wells will thus be, in ng/ml: 3160, 1000, 316, 100, 31, 10, 3.

Label eight 15 ml tubes: IH4 Control, and 1 to 7

Tube IH4 Control, will contain 10 ml PBN + 100 µl **100 X Stock of IH4 alone** @ 240 µg/ml in PBN

To perform serial dilutions of the IH4-RBD working solutions

1) Place 14.85 ml PBN in tube n°1, and 10.25 in all others.

2) Add 150 µl of 100 X IH4-RBD Stock to Tube n°1 > 4.75 µg/ml IH4-RBD

3) Use 10 ml pipet to transfer 4.75 ml to tube n°2 > 1.5 µg/ml IH4-RBD

4) Use the same 10 ml pipet to transfer 4.75 ml to tube n°3 > 470 ng/ml IH4-RBD

5) Use the same 10 ml pipet to transfer 4.75 ml to tube n°4 > 150 ng/ml IH4-RBD

6) Use the same 10 ml pipet to transfer 4.75 ml to tube n°5 > 47 ng/ml IH4-RBD

7) Use the same 10 ml pipet to transfer 4.75 ml to tube n°6 > 15 ng/ml IH4-RBD.

7) Use the same 10 ml pipet to transfer 4.75 ml to tube n°7 > 4.7 ng/ml IH4-RBD.

NB: Tube n°7 will contain 15 mls whilst the others will contain just 10.25 ml.

Once prepared, the working solutions can be kept for several weeks, or even months, at either 4°C or room temperature without any noticeable loss of activity.

HAT-field Procedure

Plate preparation:

If an adjustable pipet is available, use it to prefill the wells of a V-bottom 96-well plate with 60 µl/well of the various working solutions (Column 1: IH4 negative control, Columns 2-8 : IH4-RBD dilutions 1 to 7)

If out in the field with no access to an adjustable pipet, the reagent can be distributed just before the assay will be performed, using a plastic Pasteur pipet to dispense 2 drops (i.e. ca. 60 µl) of the working solutions in the appropriate wells, and working in increasing order of concentrations (IH4-RBD dilutions 1 to 7), and a different pipet for the IH4 nanobody negative control.

Blood collection and assay (Subject should be asked to clean hands with soap, using warm water if possible, and to dry them well so that the blood drop stays compact on the fingertip).

- Take a plastic Pasteur pipet, and make sure that you have spotted the limit between the first and second section, which corresponds to 10 µl. Take one of the small tubes with 300 µl of PBS-EDTA, and label it with the subject’s name.
- Wipe finger pulp with sterile towel/swab. Prick skin on the outside of finger pulp with disposable, single use lancet. Wipe away first drop with same sterile swab
- Massage second drop of blood to the size of a lentil
- Take up 10 µl with the Pasteur pipet (first section filled) , and transfer this blood to the small tube containing 300 µl of PBS-EDTA. Pipet up and down a couple of times, avoiding making bubbles.
- Using the same Pasteur pipet, take up all 310 µl of diluted blood, and distribute one drop into each of the 8 adjacent wells of a row, containing the IH4 control and IH4-RBD dilutions 1 to 7.
- Incubate the plate in a horizontal position for a minimum of 1hr at ambiant temperature. The red blood cells will sediment and form red dots at the bottom of the wells. Alternatively, if a plate centrifuge is available, plates can be spun at 100g for 1 minute after just 15 minutes incubation.
- Tilt plate at ca. 10° from the vertical against a well-lit white background (if possible on a lightbox, see below)
- When the RBCs have formed teardrops that reach all the way to the walls of the control wells (this should normally take less than 30 seconds), take photographs of the plate to record the results (with a smartphone, for example), making sure the camera is at least 50 cms away from the plate so that the bottom of all the wells can be clearly seen in the pictures. For this, we find that a very simple lightbox can greatly improve the ease of tilting the plates, and the quality of the pictures (see tutorial on https://youtu.be/e5zBYd19nIA)
- Scoring of the samples is done by counting the wells from the highest IH4-RBD concentration to the last well showing complete hemagglutination, i.e. a symmetrical dot with no “pointy bottom” as a result of the tilting.

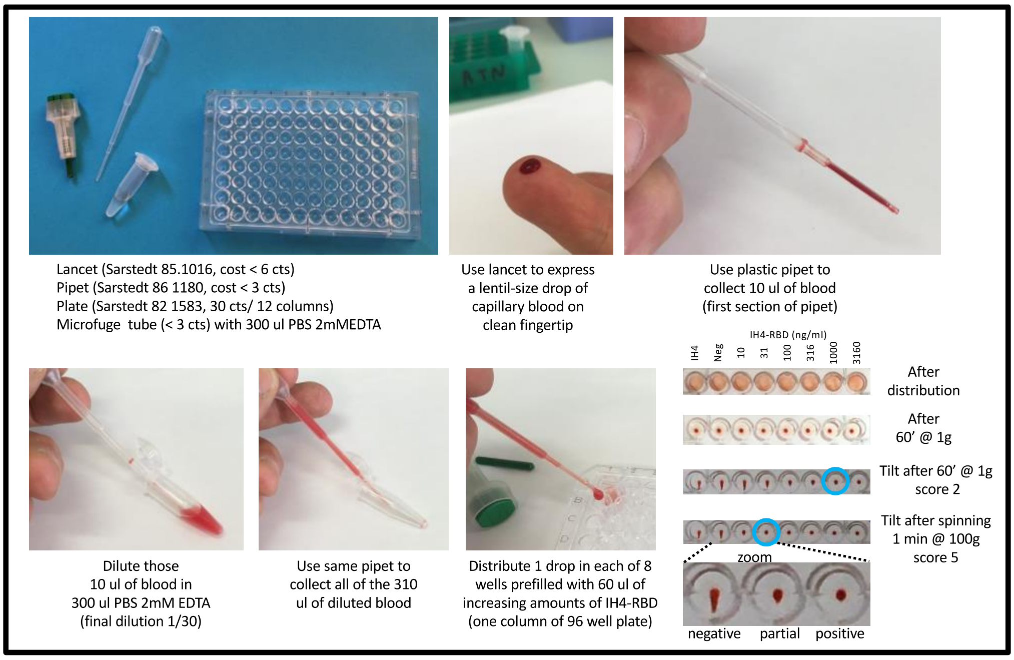

Remarks:

When performing the assay for the first time, once the 10 µl of blood have been diluted in the 300 µl PBS-EDTA, it is a good idea to start by counting the drops you will obtain by dropping them back into the tube used for the dilution, so as to ensure that you can obtain 8 drops (each drop should be just under 30 µl). If you do not, try placing the pipet at a different angle to generate smaller drops.

If a plate centrifuge is not available, longer incubations, up to 5 hours, will results in increased sensitivity. To this end, plates can be photographed after one hour, then returned to the horizontal position, and the tilting/photographing repeated at 3 and 5 hours (or other times if more convenient).

Rather than PBN, the negative control should preferably consist of the IH4 nanobody at a final concentration of 1.5 µg/ml (i.e. corresponding to the molar concentration of the IH4-RBD reagent in tube 1). Samples that show reactivity against IH4 will then have to be excluded.

Alternatively, a second row of wells containing titrations of the IH4 nanobody alone can be used to assess the plasmatic reactivity against the IH4 moiety of the IH4-RBD reagent. For this, collect 20 µl of blood by filling the plastic Pasteur pipet to the second section, and dilute this blood in 600 µl of PBS-EDTA, before using the pipet to place one drop in the 16 wells of the two rows.

For better quality photographs, it is best to use a lightbox. A very simple and cheap lightbox can be made with a cardboard box and a small lamp, such as the examples below, and in the tutorial video found at the end of this link: https://youtu.be/e5zBYd19nIA

If there are no positive samples in the set tested, the activity of the IH4-RBD can be ascertained by using a plastic Pasteur pipet to add one drop of the CR3022 mAb at 20 ng/ml to all the wells of one row. After returning the rest of the CR3022 stock to its tube, that same pipet can then be used to resuspend the RBCs in all the wells of that row, starting by the well with the lowest concentration of IH4-RBD and working towards higher concentrations, avoiding the formation of bubbles as much as possible. After a further hour of incubation, if stocks of IH4-RBD and CR3022 are fully functional, the endpoint should be around a score of 5.

Examples of lightboxes made with battery-operated LED spotlights

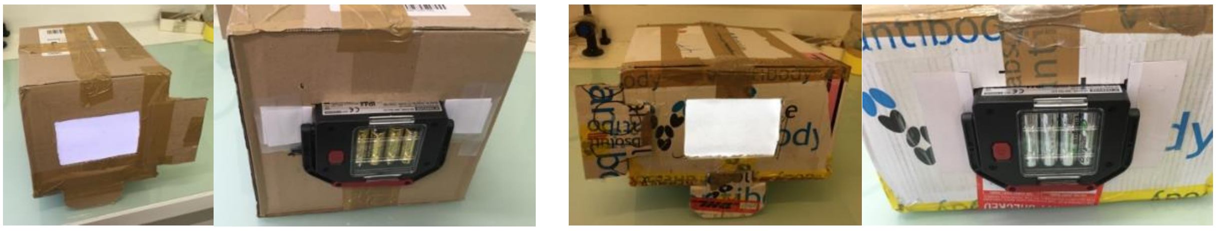

Materials:
1 small carboard box
1 sheet of white printer paper folded in two
1 small lamp with cold bulb (e.g. LED)
Sticky tape and Stanley knife or sharp scissors
Optional: Alu foil to enhance brightness and to obtain more diffuse light.

Method:
1) Use a 96-well plate to cut a window to the right size in the bottom of the box
2) The pieces of cardboard that have been cut out can be used to make 'wings' that will be taped to the bottom so that the box rests at roughly 10° (see pictures)
3) Tape the folded piece of white paper to the inside of the window cut in the bottom
4) The outer flaps at the top of the box can the wrapped in tin foil to provide a brighter a more diffuse light
5) If a 'clamp' lamp is being used, it can be good to increase the thickness of the cardboard by foling the flap over on the side where the lamp will be clamped.
6) For better pictures, turn the lights off in the room, and place the camera at least 50 cms from the plate.

Home-made trans-illuminator for taking picture of HAT plates

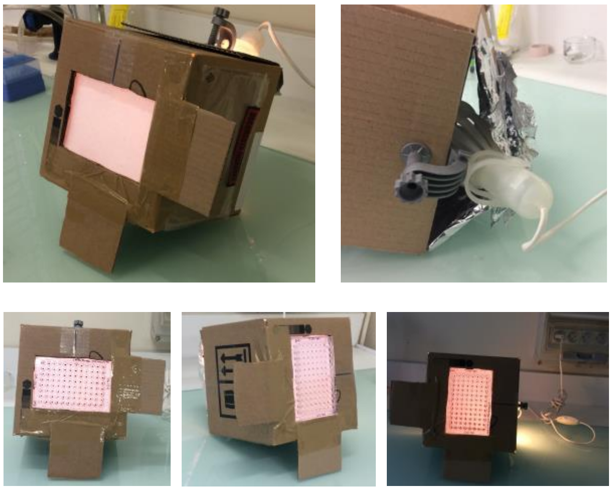

Supplement: bpac026_Supplementary_Data [file bpac026_supplementary_data.zip › Protocol for HAT-field.pdf]
