# Supplementary material for: HAT-field: a cheap, robust and quantitative Point-of-care serological test for Covid-19
Source: Biol Methods Protoc. 2022 Nov 28;7(1):bpac026. doi: 10.1093/biomethods/bpac026 (PMC9620368; doi:10.1093/biomethods/bpac026)
Supplement: bpac026_Supplementary_Data [file bpac026_supplementary_data.zip › Supplementary figures for BMP.docx]

**Supplementary figures**

**Supplementary figure 1**: Influence of the incubation times on HAT-field scores

For each of the 60 samples of the cohort of clinical whole blood samples, HAT-field was setup in three separate parallel plates, as described in Methods. The three separate plates were then submitted to the following incubation conditions:

i) Under normal gravity (1g), with tilting and taking pictures at 1, 3 and 5 hours before returning the plates to a horizontal position each time.
ii) under normal gravity for one hour, then taking pictures before and after spinning the plates for 1 minute at 100g
iii) incubating the plates for just 15 minutes horizontally at 1g before spinning it for 1 minute at 100g and then taking the pictures.

This resulted in 6 data sets, which were all compared to the values of the Jurkat-S&R-flow test (first two lines). The vertical doted red lines indicate the threshold for positive samples used for the Jurkat-S&R-flow test.

For the HAT-field test, although we recorded the partially hemagglutinated wells at the highest IH4-RBD concentration as a value of 0.5, we only consider the fully hemagglutinated wells as truly positive (score 1).

Comparison of the 3 graphs on the first line shows that, when the plates are incubated under normal gravity, the sensitivity of the detection increases with incubation times, with fewer and fewer samples identified as positives by the Jurkat-S&R-flow test (i.e. past the red line) with HAT-field scores of zero or 0.5.

The first graph on the second line is effectively a repeat of the one just above, i.e. 1g for 60 minutes. Comparison of the two shows that, although there are minor differences (i.e. the HAT-field scores of a few samples differing by just one unit), the method is highly reproducible.

The next two graphs correspond to the results obtained after spinning the plates at 100g for 1 minute after either 60 minutes (2^nd^ graph) , or after just 15 minutes (3^rd^ graph), giving results that are very comparable to one another, and to those obtained after incubating the plates for 5 hours under normal gravity.

The graphs on the third line are one on one comparisons between the sets of scores obtained under these three latter conditions, i.e. 1g for 1 hour then spin, 1g for 5 hours, or 1g for just 15 minutes before spinning. On this series of graphs, because of the discrete nature of the scales used for both the X and Y axes, many points overlap on top of one another. In such cases, their numbers are indicated by the adjacent green numbers. Although the results obtained with those three conditions are very closely related to one another, it is worthy of note that spinning the plates after just 15 minutes does result in a few samples with slightly higher scores than those obtained with the other two conditions.

**Supplementary figure 2**: Spinning of plates for plasma titrations after 60 minutes incubation under normal gravity also greatly increases sensitivity.

As can be seen by comparing the two graphs below, when performing plasma titrations using the standard HAT protocol, spinning the plate after 60 minutes of incubation at 1g (right panel) results in a marked improvement of the sensitivity, with all of the samples that were detected as positive by FACS having a HAT score of at least 1, i.e. showing full hemagglutination at 1/40, the highest plasma concentration used. The one sample which gave partial hemagglutination and FACS signal just above the positive threshold was actually most probably a false positive (see figure 6 and sample 48 in data file).

See Discussion for considerations relating to the importance of using the IH4 alone reagent as a negative control to counter the decrease in specificity linked to increased sensitivity.

**Supplementary figure 3**: Comparing scores obtained with IH4-RBD-delta to those obtained with IH4-RBD Wuhan

The above graphs were generated using:

For HAT-field, the scores obtained after spinning the plates at 100g for 1’ after 15’ incubation at 1g (columns F for Wuhan and G for Delta in the data file).

For the plasma titrations, the scores obtained after spinning the plates at 100g for 1’ after 60’ incubation at 1g (columns O for Wuhan and Q for Delta in the data file).

When several points overlap, their numbers are indicated by the adjacent green numbers.

The first graph shows that, as was seen in figure 6A with the IH4-RBD Wuhan reagent, under the conditions used in this study, plasma titrations are, overall, more sensitive than HAT-field.

The second and third graph show that, for both HAT-field and plasma titrations, the scores obtained with the IH4-RBD Delta reagent tend to be similar or lower by one or two units than those with the IH4-RBD Wuhan reagent.

The validation of the optimized HAT protocols was performed on a cohort of clinical whole blood samples collected in the course of the month of September 2021. At that time, most of the SARS-CoV-2 virus circulating in France belonged to the Delta lineage. Since those were blood samples from hospitalized patients, we were expecting to find a sizeable proportion of Delta-infected patients. Somewhat disappointingly, gathering of clinical information on the patients from which the blood samples were obtained, which took place after the experiments were carried out, actually revealed that, in our cohort of 60 samples, only 3 were from patients who had ever had a positive PCR for SARS-CoV-2.

The first patient (sample 5, red spots) had had a positive PCR in October 2020, and must thus have been infected by a viral strain different from Delta. The level of the plasmatic anti-spike antibodies measured by the Jurkat-S&R-flow test was low (specific staining signal of 61.5), dominantly of the IgG isotype (93.2 %), and higher HAT scores with the Wuhan IH4-RBD than with the Delta IH4-RBD: HAT-field : W=2, D=0 ; HAT titration: W= 5, D=2

The second patient (sample 8, yellow spots) had a positive PCR on admission, which was the day when the blood sample we used was collected, but no information on viral genotyping was available. A PCR carried out 4 days later was negative. The level of the plasmatic anti-spike antibodies measured by the Jurkat-S&R-flow test was also low (specific staining signal of 62.3), with a sizeable signal for IgMs (15% ). HAT scores were only just positive: HAT-field : W=1, D=1; HAT titration: W= 1, D=0.5. Based on this limited amount of information, no conclusion can be drawn regarding the lineage of the virus that infected that patient.

The third patient (sample 11, green spots) had a positive PCR on admission, which revealed a viral strain carrying the L452R corresponding to the delta lineage. No further information is available about the evolution of the infection. Plasmatic anti-spike antibodies were undetectable with the Jurkat-S&R-flow test (specific staining signal of 6). Among the 16 sets of HAT scores (columns B-Q), although none were clearly positive, three were recorded as partially positive, all with the IH4-RBD Delta reagent : HAT-field 60’ + spin and 15’ + spin, and titration 60’ + spin.

Although the data obtained for those three samples can only be considered as anecdotal, it is nonetheless comforting to see that they all follow the expected direction. A properly setup clinical trial, enrolling larger numbers of patients infected by various strains of the SARS-CoV-2 virus would need to be setup to formally validate the performance of the two reagents, and their capacity to follow serological responses after infection with those various strains. This was, however, far beyond the reach of this study.
