# Supplementary material for: HAT-field: a cheap, robust and quantitative Point-of-care serological test for Covid-19
Source: Biol Methods Protoc. 2022 Nov 28;7(1):bpac026. doi: 10.1093/biomethods/bpac026 (PMC9620368; doi:10.1093/biomethods/bpac026)
Supplement: bpac026_Supplementary_Data [file bpac026_supplementary_data.zip › Data cohort clinical samples .pdf]

| | HAT-field | | | | | | | | | | | | Titration on O- RBCs | | | | reactivity on IH4 alone | | | | Jurkat S&R flow results | | | | | | | | Clinical data | |
|---|---|---|---|---|---|---|---|---|---|---|---|---|---|---|---|---|---|---|---|---|---|---|---|---|---|---|---|---|---|---|
| | Wuhan | | Delta | | Wuhan | Delta | Wuhan | | | Delta | | | Scores titration Wuhan | | Scores titration Delta | | HAT-field scores | | titrations on 0.5 ug/ml | | | | | | | | | | | |
| Sample n° | 60 min 1g | + spin | 60 min 1g | + spin | Spin @ 15 min | Spin @ 15 min | 1 hour | 3 hours | 5 hours | 1 hour | 3 hours | 5 hours | 1 hour | + spin | 1 hour | + spin | 60 min | + spin | 60 min | + spin | J-S&R 488 GAM | G | A | M | G+A+M | %G | %A | %M | Gender | Covid / PCR histories |
| 1 | 3 | 5 | 2 | 4 | 5 | 4 | 3 | 4 | 5 | 3 | 3 | 4 | 3 | 5 | 3 | 5 | | | | | 158.79 | 172.32 | 4.61 | 1.26 | 178.2 | 96.7 | 2.6 | 0.7 | F | |
| 2 | 0 | 0.5 | 0 | 0 | 0.5 | 0 | 0.5 | 0.5 | 0.5 | 0 | 0 | 0.5 | 1 | 2 | 0.5 | 2 | | | | | 38.1 | 42.29 | 3.01 | 0.6 | 45.9 | 92.1 | 6.6 | 1.3 | F | |
| 3 | 4 | 4 | 3 | 3 | 4 | 3 | 3 | 4 | 4 | 3 | 3 | 3 | 7 | 8 | 7 | 8 | | | | | 215.78 | 190.72 | 16.58 | 92.68 | 300.0 | 63.6 | 5.5 | 30.9 | M | |
| 4 | 0 | 0 | 0 | 0 | 0 | 0 | 0 | 0 | 0 | 0 | 0 | 0 | 0 | 0.5 | 0 | 0.5 | | | | | 2.7 | 2.58 | 1.12 | 2.47 | 6.2 | | | | M | |
| 5 | 1 | 2 | 0 | 0 | 2 | 0 | 0.5 | 1 | 1 | 0 | 0 | 0 | 3 | 5 | 1 | 2 | | | | | 61.5 | 64.1 | 2.51 | 2.18 | 68.8 | 93.2 | 3.6 | 3.2 | M | Covid in oct 2020 (PCR pos) |
| 6 | 4 | 6 | 4 | 5 | 6 | 5 | 4 | 5 | 6 | 4 | 5 | 6 | 5 | 8 | 6 | 8 | | | 2 | 4 | 907.4 | 1066.14 | 27.12 | 9.1 | 1102.4 | 96.7 | 2.5 | 0.8 | F | |
| 7 | 0.5 | 4 | 0 | 3 | 4 | 4 | 0.50 | 2 | 4 | 0 | 2 | 4 | 3 | 5 | 2 | 4 | | | | | 355.65 | 380.74 | 8.34 | 5.53 | 394.6 | 96.5 | 2.1 | 1.4 | F | |
| 8 | 0 | 1 | 0 | 0.5 | 1 | 1 | 0 | 0.5 | 0.5 | 0 | 0.5 | 1 | 0.5 | 1 | 0 | 0.5 | | | | | 62.3 | 67.7 | 2.38 | 12.4 | 82.5 | 82.1 | 2.9 | 15.0 | F | pos PCR on hosp entry, neg on day 4 |
| 9 | 5 | 6 | 3 | 6 | 6 | 6 | 5 | 6 | 6 | 4 | 6 | 7 | 6 | 8 | 5 | 7 | | | | | 981.27 | 1173.48 | 53.72 | 13.02 | 1240.2 | 94.6 | 4.3 | 1.0 | M | |
| 10 | 3 | 6 | 3 | 5 | 7 | 6 | 3 | 5 | 5 | 3 | 4 | 5 | 2 | 4 | 2 | 4 | | | | | 569.08 | 693.8 | 52.4 | 1.48 | 747.7 | 92.8 | 7.0 | 0.2 | M | |
| 11 | 0 | 0 | 0 | 0.5 | 0 | 0.5 | 0 | 0 | 0 | 0 | 0 | 1 | 0 | 0 | 0 | 0.5 | | | | | 6 | 6.55 | 0.95 | 0.51 | 8.0 | | | | F | pos PCR, with L452R mutation |
| 12 | 6 | 7 | 6 | 7 | 7 | 7 | 6 | 7 | 7 | 6 | 7 | 7 | 5 | 7 | 6 | 8 | 0 | 1 | 0.5 | 1 | 1401.39 | 1643.68 | 208.3 | 8.94 | 1860.9 | 88.3 | 11.2 | 0.5 | M | |
| 13 | 1 | 3 | 0 | 1 | 2 | 2 | 1 | 2 | 3 | 0 | 1 | 2 | 2 | 4 | 2 | 3 | | | | | 195.9 | 242 | 20.03 | 2.8 | 264.8 | 91.4 | 7.6 | 1.1 | F | |
| 14 | 4 | 5 | 3 | 4 | 5 | 4 | 3 | 4 | 5 | 3 | 4 | 5 | 5 | 7 | 5 | 7 | | | | | 862 | 962.53 | 117.14 | 7.33 | 1087.0 | 88.5 | 10.8 | 0.7 | M | |
| 15 | 0 | 0 | 0 | 0 | 0 | 0 | 0 | 0 | 0 | 0 | 0 | 0 | 0 | 0 | 0 | 0 | | | | | 2 | 1.38 | 0.56 | -0.12 | 1.8 | | | | M | |
| 16 | 0 | 2 | 0 | 1 | 2 | 1 | 0 | 0.5 | 2 | 0 | 0 | 1 | 2 | 4 | 2 | 3 | | | | | 614.49 | 716.92 | 63.03 | 6.12 | 786.1 | 91.2 | 8.0 | 0.8 | F | |
| 17 | 0 | 0 | 0 | 0 | 0 | 0 | 0 | 0 | 0 | 0 | 0 | 0 | 0.5 | 1 | 0.5 | 1 | | | 0 | 1 | 4.81 | 6.68 | 0.81 | -0.17 | 7.3 | | | | M | |
| 18 | 5 | 6 | 4 | 5 | 6 | 5 | 5 | 6 | 7 | 4 | 5 | 6 | 5 | 7 | 5 | 7 | | | | | 1338.19 | 1519.39 | 96.39 | 2.43 | 1618.2 | 93.9 | 6.0 | 0.2 | M | |
| 19 | 3 | 3 | 2 | 2 | 3 | 2 | 2 | 3 | 3 | 2 | 2 | 3 | 5 | 7 | 5 | 6 | 3 | 3 | 5 | 6 | 7.1 | 17.7 | 0.83 | -13.34 | 5.2 | | | | M | |
| 20 | 0 | 0.5 | 0 | 0.5 | 1 | 0.5 | 0 | 0 | 0 | 0 | 0 | 0 | 1 | 3 | 1 | 2 | | | | | 125.99 | 114.68 | 84.24 | 0.46 | 199.4 | 57.5 | 42.3 | 0.2 | F | |
| 21 | 5 | 6 | 4 | 6 | 7 | 6 | 5 | 6 | 6 | 4 | 5 | 7 | 5 | 7 | 5 | 7 | | | | | 1518.52 | 1977.44 | 48.5 | 2.46 | 2028.4 | 97.5 | 2.4 | 0.1 | M | |
| 22 | 1 | 3 | 0 | 0.5 | 3 | 1 | 1 | 2 | 3 | 0 | 0 | 1 | 3 | 5 | 2 | 2 | | | | | 40.76 | 40.25 | 34.41 | 0.02 | 74.7 | 53.9 | 46.1 | 0.0 | F | |
| 23 | 3 | 5 | 3 | 4 | 5 | 4 | 3 | 4 | 5 | 3 | 4 | 5 | 4 | 6 | 4 | 5 | | | | | 705.9 | 879.85 | 8.41 | 1.87 | 890.1 | 98.8 | 0.9 | 0.2 | F | |
| 24 | 0 | 0.5 | 0 | 0.5 | 0.5 | 0.5 | 0 | 0 | 1 | 0 | 0 | 0.5 | 2 | 4 | 1 | 3 | | | 0.5 | 1 | 318.73 | 320.2 | 121.44 | -0.11 | 441.5 | 72.5 | 27.5 | 0.0 | M | |
| 25 | 1 | 2 | 0 | 0 | 2 | 0 | 1 | 1 | 2 | 0 | 0 | 0 | 3 | 4 | 1 | 2 | | | | | 87.95 | 107.31 | 6.93 | -0.47 | 113.8 | 94.3 | 6.1 | -0.4 | M | |
| 26 | 1 | 4 | 1 | 3 | 4 | 3 | 1 | 3 | 4 | 1 | 2 | 3 | 2 | 4 | 2 | 3 | | | | | 285.97 | 324.66 | 8.35 | 1.21 | 334.2 | 97.1 | 2.5 | 0.4 | F | |
| 27 | 7 | 7 | 7 | 7 | 7 | 7 | 7 | 7 | 7 | 7 | 7 | 7 | 5 | 8 | 6 | 8 | | | | | 2380.5 | 2648.33 | 77.85 | 57.1 | 2783.3 | 95.2 | 2.8 | 2.1 | F | |
| 28 | 0 | 0 | 0 | 0 | 0 | 0 | 0 | 0 | 0 | 0 | 0 | 0 | 1 | 2 | 0.5 | 1 | | | | | 46.96 | 48.95 | 9 | 5.63 | 63.6 | 77.0 | 14.2 | 8.9 | M | |
| 29 | 0 | 0 | 0 | 0 | 0 | 0 | 0 | 0 | 0 | 0 | 0 | 0 | 0.5 | 1 | 0 | 0.5 | | | | | 15.77 | 17.49 | 8.09 | 0.33 | 25.9 | 67.5 | 31.2 | 1.3 | M | |
| 30 | 0 | 0.5 | 0 | 0 | 0.5 | 0 | 0 | 0 | 0 | 0 | 0 | 0 | 2 | 3 | 2 | 3 | | | | | 201.07 | 244.52 | 4.3 | 0.4 | 249.2 | 98.1 | 1.7 | 0.2 | M | |
| 31 | 5 | 6 | 5 | 6 | 6 | 6 | 5 | 6 | 7 | 5 | 6 | 6 | 5 | 7 | 6 | 8 | | | | | 639.8 | 808.96 | 20.27 | 7.3 | 836.5 | 96.7 | 2.4 | 0.9 | F | |
| 32 | 0 | 1 | 0 | 0 | 1 | 0 | 0 | 0 | 0 | 0 | 0 | 0 | 0.5 | 1 | 0 | 0.5 | | | | | 55.4 | 64.96 | 6.78 | -0.89 | 70.9 | 91.7 | 9.6 | -1.3 | M | |
| 33 | 2 | 5 | 2 | 4 | 5 | 5 | 2 | 4 | 5 | 2 | 3 | 5 | 3 | 5 | 3 | 5 | | | | | 651.5 | 741.8 | 24.21 | -0.85 | 765.2 | 96.9 | 3.2 | -0.1 | F | |
| 34 | 6 | 7 | 6 | 6 | 7 | 7 | 5 | 7 | 7 | 6 | 7 | 7 | 7 | 8 | 7 | 8 | | | | | 1850.82 | 2295.06 | 97.08 | 59.12 | 2451.3 | 93.6 | 4.0 | 2.4 | F | |
| 35 | 0 | 0 | 0 | 0 | 0 | 0 | 0 | 0 | 0 | 0 | 0 | 0 | 0 | 0.5 | 0 | 0 | | | | | 3.11 | 4.31 | 2.3 | -1.44 | 5.2 | | | | M | |
| 36 | 4 | 6 | 4 | 6 | 6 | 6 | 4 | 6 | 6 | 4 | 5 | 6 | 4 | 6 | 4 | 6 | | | | | 892.12 | 1022.68 | 10.7 | 2.02 | 1035.4 | 98.8 | 1.0 | 0.2 | M | |
| 37 | 5 | 6 | 4 | 6 | 6 | 6 | 5 | 6 | 6 | 4 | 5 | 6 | 4 | 7 | 5 | 6 | | | | | 1191.18 | 1434.3 | 30.3 | 7.2 | 1471.9 | 97.4 | 2.1 | 0.5 | M | |
| 38 | 0 | 0 | 0 | 0 | 0.5 | 0 | 0 | 0.5 | 0.5 | 0 | 0 | 0.5 | 0.5 | 1 | 0 | 1 | | | | | 66.32 | 82.9 | 2.4 | 2.7 | 88.1 | 94.2 | 2.7 | 3.1 | M | |
| 39 | 2 | 4 | 2 | 3 | 5 | 3 | 2 | 3 | 4 | 2 | 3 | 6 | 2 | 3 | 1 | 2 | 0 | 1 | 1 | 2 | 265.7 | 324.3 | 27.8 | 0.6 | 352.7 | 91.9 | 7.9 | 0.2 | M | |
| 40 | 0.5 | 2 | 1 | 2 | 4 | 2 | 0.5 | 2 | 2 | 1 | 1 | 2 | 2 | 4 | 2 | 3 | | | | | 296.58 | 365.3 | 15.4 | 0.3 | 381.0 | 95.9 | 4.0 | 0.1 | F | |
| 41 | 0 | 0 | 0 | 0.5 | 0.5 | 0.5 | 0 | 0 | 0 | 0 | 0 | 0.5 | 0.5 | 1 | 0.5 | 1 | | | | | 106.9 | 122.0 | 32.7 | -0.3 | 154.5 | 79.0 | 21.2 | -0.2 | F | |
| 42 | 0 | 1 | 0 | 1 | 2 | 1 | 0 | 1 | 2 | 0 | 0 | 1 | 2 | 4 | 1 | 3 | | | | | 236.46 | 287.6 | 11.8 | 1.1 | 300.4 | 95.7 | 3.9 | 0.4 | M | |
| 43 | 5 | 5 | 4 | 5 | 7 | 6 | 5 | 6 | 6 | 4 | 5 | 6 | 4 | 5 | 4 | 5 | | | | | 485.3 | 573.2 | 19.5 | 4.8 | 597.5 | 95.9 | 3.3 | 0.8 | F | |
| 44 | 5 | 6 | 4 | 5 | 6 | 5 | 5 | 5 | 6 | 4 | 5 | 5 | 6 | 8 | 6 | 7 | | | | | 1072.98 | 1443.3 | 82.8 | 46.9 | 1573.0 | 91.8 | 5.3 | 3.0 | M | |
| 45 | 1 | 2 | 0.5 | 2 | 3 | 3 | 1 | 2 | 3 | 0.5 | 2 | 3 | 3 | 5 | 2 | 4 | | | | | 542.57 | 735.6 | 8.7 | 1.7 | 746.0 | 98.6 | 1.2 | 0.2 | M | |
| 46 | 2 | 4 | 2 | 3 | 5 | 4 | 2 | 4 | 4 | 2 | 3 | 4 | 4 | 5 | 4 | 5 | | | 0.5 | 1 | 481.13 | 595.9 | 5.4 | 2.2 | 603.5 | 98.7 | 0.9 | 0.4 | M | |
| 47 | 0 | 0 | 0 | 0 | 2 | 2 | 0 | 0 | 0 | 0 | 0 | 0 | 1 | 2 | 1 | 2 | | | | | 116.7 | 145.7 | 1.5 | 1.0 | 148.2 | 98.3 | 1.0 | 0.7 | F | |
| 48 | 0 | 0.5 | 0 | 0.5 | 0.5 | 0.5 | 0 | 0.5 | 0.5 | 0 | 0 | 0.5 | 0.5 | 0.5 | 0.5 | 0.5 | | | 2 | 3 | 44.85 | 53.7 | 3.2 | 7.7 | 64.6 | 83.2 | 4.9 | 11.9 | M | |
| 49 | 0 | 0.5 | 0 | 0 | 0.5 | 0 | 0 | 0 | 0 | 0 | 0 | 0 | 1 | 2 | 0 | 0.5 | | | | | 17.1 | 20.4 | 2.1 | 0.5 | 23.0 | 88.9 | 8.9 | 2.1 | M | |
| 50 | 0 | 0.5 | 0 | 1 | 0.5 | 1 | 0 | 0 | 0 | 0 | 0 | 0.5 | 2 | 3 | 2 | 3 | | | 0.5 | 1 | 93.89 | 107.3 | 1.8 | 2.4 | 111.5 | 96.3 | 1.6 | 2.2 | F | |
| 51 | 0 | 0.5 | 0 | 1 | 0.5 | 1 | 0 | 0 | 0 | 0 | 1 | 0.5 | 1 | 2 | 0.5 | 1 | | | | | 20.74 | 23.1 | 1.8 | 2.3 | 27.1 | 85.2 | 6.5 | 8.3 | F | |
| 52 | 4 | 4 | 2 | 3 | 4 | 3 | 3 | 4 | 4 | 2 | 3 | 3 | 3 | 4 | 3 | 3 | 0 | 1 | 2 | 2 | 275.1 | 294.9 | 10.7 | 11.8 | 317.4 | 92.9 | 3.4 | 3.7 | M | |
| 53 | 2 | 4 | 1 | 3 | 4 | 3 | 2 | 3 | 4 | 1 | 3 | 4 | 3 | 6 | 4 | 5 | | | | | 224.83 | 223.7 | 100.5 | 0.5 | 324.6 | 68.9 | 30.9 | 0.1 | M | |
| 54 | 4 | 6 | 2 | 5 | 5 | 4 | 4 | 5 | 5 | 3 | 4 | 4 | 4 | 7 | 5 | 7 | | | | | 567.1 | 716.5 | 6.6 | -0.3 | 722.8 | 99.1 | 0.9 | 0.0 | M | |
| 55 | 0.5 | 2 | 1 | 2 | 3 | 2 | 0.5 | 1 | 2 | 1 | 1 | 2 | 2 | 4 | 2 | 3 | | | 0 | 1 | 287.5 | 310.8 | 312.8 | 0.3 | 623.9 | 49.8 | 50.1 | 0.0 | F | |
| 56 | 0 | 1 | 0 | 0.5 | 1 | 1 | 0 | 0.5 | 0.5 | 0 | 1 | 1 | 1 | 2 | 1 | 1 | | | | | 82.8 | 101.4 | 21.6 | -1.1 | 121.9 | 83.2 | 17.7 | -0.9 | M | |
| 57 | 7 | 7 | 6 | 7 | 7 | 7 | 7 | 7 | 7 | 6 | 7 | 7 | 7 | 8 | 8 | 8 | | | | | 1279.28 | 1765.8 | 21.8 | 55.5 | 1843.2 | 95.8 | 1.2 | 3.0 | M | |
| 58 | 0 | 0 | 0.5 | 1 | 1 | 1 | 0 | 0 | 0 | 0 | 0 | 0 | 0 | 0 | 0 | 0.5 | | | | | 2.6 | 5.3 | 1.9 | 0.1 | 7.3 | | | | M | |
| 59 | 0.5 | 3 | 0.5 | 2 | 3 | 3 | 0.5 | 2 | 3 | 0.5 | 2 | 2 | 2 | 3 | 2 | 3 | 1 | 1 | 0.5 | 2 | 165.94 | 199.9 | 6.8 | 0.3 | 207.0 | 96.6 | 3.3 | 0.2 | F | |
| 60 | 4 | 6 | 4 | 6 | 6 | 6 | 4 | 6 | 6 | 4 | 5 | 5 | 5 | 7 | 5 | 7 | | | | | 864.11 | 1190.2 | 13.2 | 8.2 | 1211.6 | 98.2 | 1.1 | 0.7 | F | |
